# Antibacterial and Synergistic Activity of Pentacyclic Triterpenoids Isolated from *Alstonia scholaris*

**DOI:** 10.3390/molecules21020139

**Published:** 2016-01-25

**Authors:** Chao-Min Wang, Hsiao-Ting Chen, Zong-Yen Wu, Yun-Lian Jhan, Ching-Lin Shyu, Chang-Hung Chou

**Affiliations:** 1 Research Center for Biodiversity, China Medical University, Taichung, 40402, Taiwan; wangchaomin@mail.cmu.edu.tw (C.-M.W.); shine_5201314@yahoo.com.tw (H.-T.C.); ah_giu@yahoo.com.tw (Y.-L.J.); 2 Department of Veterinary Medicine, College of Veterinary Medicine, National Chung-Hsing University, Taichung, 40402, Taiwan; zongyan630@yahoo.com.tw (Z.-Y.W.); clshyu@nchu.edu.tw (C.-L.S.)

**Keywords:** *Alstonia scholaris*, triterpenoid, antibacterial, synergistic, ursolic acid, MRSA

## Abstract

(1) Background: *Alstonia scholaris* (Apocynaceae) is an important medicinal plant that has been historically used in “Dai” ethnopharmacy to treat infectious diseases in China. Although various pharmacological activities have been reported, the antimicrobial constitutes of *A. scholaris* have not yet been identified. The objective of this study is to evaluate the antibacterial constitutes from the leaf extract of *A. scholaris* and to assess the synergistic effects of isolated compounds with antibiotics against bacterial pathogens.; (2) Methods: The chemical constitutes isolated from the leaf extract of *A. scholaris* were structurally identified by NMR. The antibacterial and synergistic effect of compounds was assessed by calculating the minimal inhibitory concentration (MIC), checkerboard dilution test, and time-kill assay.; (3) Results: Six pentacyclic triterpenoids were structurally identified as (**1**) lupeol, (**2**) betulin, (**3**) 3-hydroxy-11-ursen-28,13-olide, (**4**) betulinic acid, (**5**) oleanolic acid and (**6**) ursolic acid. Both oleanolic and ursolic acid showed antibacterial activity but were limited to Gram-positive bacteria. Ursolic acid showed a synergistic effect with ampicillin and tetracycline against both *Bacillus cereus* and *S. aureus*.; (4) Conclusion: These findings reflect that pentacyclic triterpenoids are the antibacterial chemicals in *A. scholaris*. The ability of ursolic acid to enhance the activity of antibiotics can constitute a valuable group of therapeutic agents in the future.

## 1. Introduction

The *Alstonia* belonging to family Apocynaceae is widely distributed in the tropical regions of Africa and Asia. *Alstonia scholaris* (L.) R. Br., commonly called blackboard tree, devil tree or milkwood pine, is a tropical evergreen tree native to South and Southeast Asia. Initially it was called *Echites scholaris* and the name of the scholaris species was derived from the usage of its wood as a blackboard for schools in Southeast Asia [1]. In China, the leaves of *A. scholaris* have been historically used in ‘‘Dai” ethnopharmacy to treat chronic respiratory diseases and infectious diseases [2]. On the other hand, *A. scholaris* are also used in traditional medicinal systems of India, Thailand, Malaysia, Philippines, Africa and Australia [3]. The chemical constituents of *Alstonia* sp. have been extensively investigated and nearly 400 compounds were reported in *Alstonia* genus [4,5,6]. Extracts of *A. scholaris* processes a wide spectra of pharmacological activities including anti-plasmodial [7], hepatoprotective [8], anti-cancer [9], anti-inflammatory and analgesic effects [10], anti-diabetic and anti-hyperlipidemic [4,11], anti-tussive, anti-asthmatic and expectorant activities [2]. Although recent studies on antimicrobial screening of *A. scholaris* demonstrate the potential antimicrobial activity of *A. scholaris* [12], the potent chemical constitutes with exact effective concentration have not yet been identified.

In the past decades, the antibiotic resistance of bacteria has emerged as a serious global problem in human and veterinary medicine. The abuse of antibiotics for non-prescription application has accelerated the generation of superbacteria that has become a critical issue. Thus, the development of new antibiotics or therapeutic strategies against multi-drug resistant bacteria is urgently needed. To pursue next generation therapeutics, several strategies, including isolating antimicrobial peptides from microorganisms, metal nanoparticles application and natural products from plant derived compounds, such as phenylpropanoids, flavonoids and triterpenoids were necessary [13,14,15]. The plant-derived chemicals enhancing the bacteria susceptibility to other antibiotics have received increasing attention. Additionally, several terpenoids, diterpenoids and sesquiterpenoids were also found to act synergistically with classes of antibiotics, indicating that plant-derived chemicals have the potential to be used as therapeutics to enhance the activity of antibiotic against multidrug-resistant pathogens [14,16,17].

Therefore, the aim of this study was to further investigate the antibacterial constitutes from the leaf extracts of *A. scholaris* against several pathogenic bacteria. Moreover, the synergistic interactions of ampicillin and tetracycline in combination with effective compounds against bacterial pathogens were also conducted. It is suggested that these compounds might have the potentiality for application in synergistic therapeutics with antibiotics in the future.

## 2. Results

### 2.1. Isolation and Identification of Triterpenoids from A. scholaris

**Figure 1:**
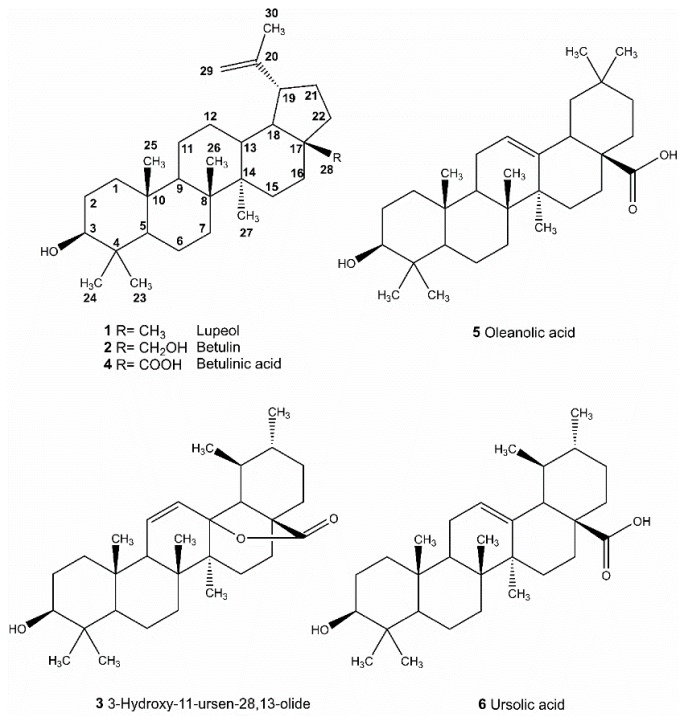
Pentacyclic triterpenoids isolated and identified from *A. scholaris*.

The antibacterial constitutes of most effective fractions in EtOAc portion (fraction EA-8 and EA-12) (Table 1) were isolated by using column chromatography to obtain six pure compounds: compound **1** (5.3 mg), **2** (32.4 mg), **3** (23.5 mg), **4** (12.6 mg), **5** (40.7 mg) and **6** (102.2 mg). Purified compounds were subjected to spectroscopic identification by using ^1^H-NMR and ^13^C-NMR (Bruker Avance 400) and Mass (Bruker Daltonics Esquire HCT). Chemical structures of compounds **1**–**6** were illustrated in Figure 1.

**Table 1:** Antibacterial activities of chemical fractions from the leaf extract of *A. scholaris*.

Pathogens	Fractions					
	Hex *	EA	BuOH	Aq	EA-8	EA-12
Methicillin-sensitive *S. aureus*	0 ^#^	10	8	7	8	12
*Enterococcus faecalis*	0	10	8	8	10	12
*Listeria monocytogenes*	0	12	10	7	8	10
*Bacillus cereus*	0	16	12	7	14	22
*Escherichia coli*	0	0	0	0	0	0
*Salmonella enterica*	0	0	0	0	0	0
*Pseudomonas aeruginosa*	0	0	0	0	0	0

* Hex: hexane layer; EA: ethyl acetate layer; BuOH: butanol layer; Aq: aqueous layer; ^#^ Inhibition zone diameters (mm).

### 2.2. The Minimal Inhibitory Concentrations (MICs) of Triterpenoids on Bacterial Pathogens

As shown in Table 2, most strains except *S. aureus*, *P. aeruginosa* and *B. cereus* were susceptible to ampicillin. The MICs of ampicillin were considerably higher at 128 μg/mL for *B. cereus* and 512 μg/mL for highly resistant *S. aureus* and *P. aeruginosa*. In addition, only *P. aeruginosa* was resistant to tetracycline at concentration of 32 μg/mL. The MICs for other bacterial pathogens were between 1 to 4 μg/mL of ampicillin and 0.5 to 8 μg/mL of tetracycline, respectively. In comparison, no significant differences were observed in the susceptibility of all Gram-negative pathogens to natural triterpenoids. *L. monocytogenes* was sensitive to oleanolic acid and ursolic acid with MICs of 8 μg/mL and 2 μg/mL, respectively. In addition, *B. cereus* was also sensitive to oleanolic acid and ursolic acid with MICs of 16 μg/mL and 8 μg/mL, respectively. *E. faecalis* characterized by high sensitivity to the triterpenoids tested, with MICs of 4 μg/mL for oleanolic acid and 1 μg/mL for ursolic acid, respectively. Particularly, only ursolic acid exhibited antibacterial activity against Methicillin-sensitive *S. aureus* (MSSA) and Methicillin-resistant *S. aureus* (MRSA).

**Table 2:** The minimum inhibitory concentration of antibiotics and natural triterpenoids for different bacterial pathogens.

Pathogens	Minimum Inhibitory Concentration (μg/mL)							
	Antibiotics and Triterpenoids							
	Ap *	Tet	1	2	3	4	5	6
Methicillin-sensitive *S. aureus*	16	8	>128	>128	>128	>128	>128	**16**
Methicillin-resistant *S. aureus*	512	8	>128	>128	>128	>128	>128	**64**
*Enterococcus faecalis*	2	4	**128**	>128	**128**	**128**	**4**	**1**
*Listeria monocytogenes*	1	2	>128	>128	>128	>128	**8**	**2**
*Bacillus cereus*	128	4	>128	>128	**128**	**128**	**16**	**8**
*Escherichia coli*	4	0.5	>128	>128	>128	>128	>128	>128
*Salmonella enterica*	1	8	>128	>128	>128	>128	>128	>128
*Pseudomonas aeruginosa*	512	32	>128	>128	>128	>128	>128	>128

* Ap: ampicillin; Tet: tetracycline; Compound **1**: lupeol; **2**: betulin; **3**: 3-hydroxy-11-ursen-28,13-olide; **4**: betulinic acid; **5**: oleanolic acid; **6**: ursolic acid.

### 2.3. Evaluation of Synergistic Effects

In the presence of triterpenoids combination with ampicillin against *B. cereus* and *S. aureus*, the interaction data in the form of the fractional inhibitory concentration indices (FICIs) are listed in Table 3. The meaning of FICI as synergistic effect (≤0.5), additional or indifference effect (0.5–4) and antagonism effect (≥4), were described previously [18]. Both oleanolic acid and ursolic acid were synergistic with ampicillin and tetracycline against *B. cereus* with FICI values of 0.281 (UA + Amp), 0.25 (UA + Tet), 0.188 (OA + Amp) and 0.078 (OA + Tet), respectively (Table 3). These values were below 0.5 and indicative of synergistic effect. *B. cereus* was most susceptible to combinations of oleanolic acid with tetracycline. Ursolic acid also displayed synergistic activity against *L. monocytogenes* with FICI values of 0.125. However, oleanolic acid did not exhibit synergy with any of tested antibiotics against *E. faecalis* and *L. monocytogenes*. In the assay of triterpenoids in combination with ampicillin against *E. faecalis* and *L. monocytogenes*, none of the triterpenoids exhibited a synergistic effect with ampicillin.

**Table 3:** Fractional inhibitory concentration indices (FICIs) calculated based on checkerboard assay indicating the interaction of triterpenoids with antibiotics against Gram-positive pathogens.

Pathogens	Agents	FIC_A_	FIC_B_	FICI	Outcome
MSSA	UA + Amp	0.25	0.125	0.375	Synergy
	UA + Tet	0.125	0.0625	0.188	Synergy
MRSA	UA + Amp	0.25	0.125	0.375	Synergy
	UA + Tet	0.0625	0.031	0.093	Synergy
*B. cereus*	UA + Amp	0.25	0.031	0.281	Synergy
	UA + Tet	0.125	0.125	0.25	Synergy
	OA + Amp	0.125	0.0625	0.188	Synergy
	OA + Tet	0.015	0.062	0.078	Synergy
*E. faecalis*	UA + Amp	0.5	0.25	0.725	Indifferent
	UA + Tet	0.125	0.5	0.625	Indifferent
	OA + Amp	1	1	2	Indifferent
	OA + Tet	1	1	2	Indifferent
*L. monocytogenes*	UA + Amp	0.5	0.0625	0.563	Indifferent
	UA + Tet	0.0625	0.0625	0.125	Synergy
	OA + Amp	1	1	2	Indifferent
	OA + Tet	1	0.5	1.5	Indifferent

### 2.4. Time-Kill Curve Assay

**Figure 2:**
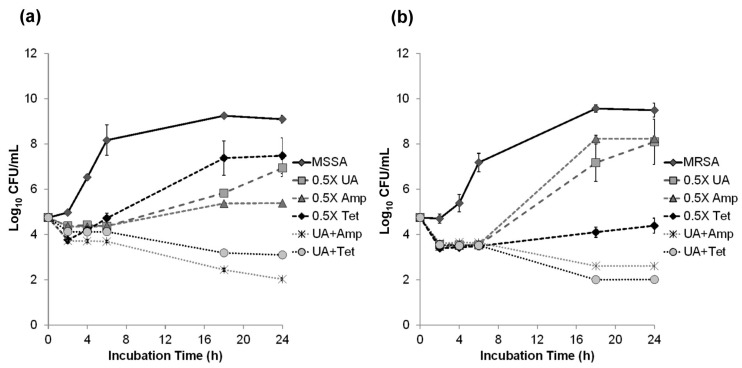

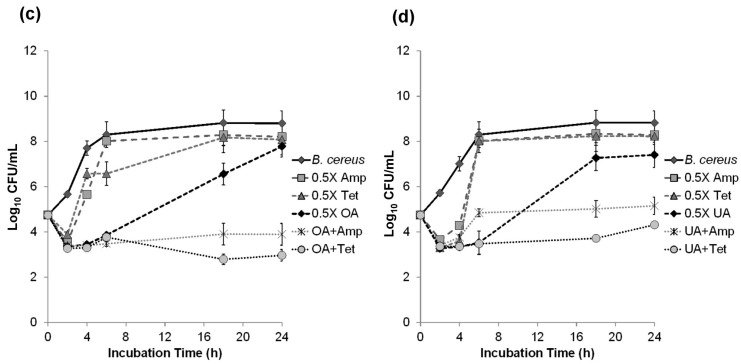
Time-kill curves of MSSA (**a**); MRSA (**b**) and *B. cereus* (**c**,**d**) with 1/2 MICs of antibiotics and triterpenoids. Ampicillin (Amp); Tetracycline (Tet); Ursolic acid (UA); Oleanolic acid (OA).

Time-kill assay was conducted to examine the synergistic effect of the combinations of triterpenoids and ampicillin on *B. cereus* and *S. aureus*. A reduction of >2 log_10_ in the cell count obtained in the presence of triterpenoids and antibiotics was interpreted as synergy [19]. After 24 h incubation, the combination of 1/2 MICs of ursolic acid with antibiotics remarkably inhibited the growth of MSSA and MRSA (Figure 2a,b). The combination of 8 μg/mL oleanolic acid with 64 μg/mL ampicillin or 2 μg/mL tetracycline also produced decrease in colony-forming unit (CFU) of >2 log_10_ against *B. cereus* (Figure 2c). A lesser effect of 4 μg/mL ursolic acid with 64 μg/mL ampicillin was observed against *B. cereus*. In summary, the results confirmed the synergy of ursolic acid with antibiotics against MRSA, MSSA and *B. cereus* (Figure 2).

## 3. Discussion

It is clear that methicillin-resistance *Staphylococcus aureus* (MRSA) has undoubtedly caused serious wounds and other infections during the last decade [20]. MRSA rates continue to increase rapidly in many regions, and there is a dynamic spread of strains across the globe [21]. Compared to MRSA, *B. cereus* produced a potent β-lactamase conferring marked resistance to β-lactam antibiotics [22]. Resistance of *B. cereus* to erythromycin, tetracycline, and carbapenem has also been reported [23]. In the rice-consuming countries, including Taiwan, Japan and Korea, the number of outbreaks caused by *B. cereus* is particularly higher than other countries [24,25,26,27]. Therefore, in the empirical management of *B. cereus* and MRSA infection, antimicrobials noted to be effective while awaiting antimicrobial susceptibility results for the isolates. To address this issue, several processes including enhancement of the antibacterial activity of antibiotics by plant-derived compounds have been undertaken to provide some guidance while awaiting isolate-specific susceptibility data [13,28,29].

Triterpenes existed abundantly in the plant and their biological activities, such as anti-HIV, antimicrobial, allelopathy, anti-tumor and anti-cancer activities were proposed [14,30,31,32,33]. In this study, ursolic acid showed specific antibacterial activity against *B. cereus* with MIC values of 2 μg/mL. The findings of this study indicated natural triterpenoids exhibited potential activity against Gram-positive pathogens, particularly *B. cereus* and *S. aureus*. However, none of the Gram-negative bacteria were sensitive to natural triterpenoids in this study.

Ampicillin and tetracycline, agents of β-lactam and anti-translation classes, are inhibitors of the cell wall and protein biosynthesis. β-lactams block enzymes involved in the cell wall synthesis while tetracyclines prevent the binding of aminoacyl-tRNA to the A site on 30S ribosome [34]. The synergistic effect of triterpenoids and both ampicillin and tetracycline were also conducted in this study. As the structures of the pentacyclic triterpenoids are different from both these antibiotic agents, the pathways in the antimicrobial activity of ursolic acid may have a novel mechanism or target in *B. cereus* and *S. aureus*.

Studies on antimicrobial mechanisms of oleanolic acid and ursolic acid demonstrated that both of the pentacyclic triterpenoids can modulate resistance to two β-lactam antibiotics, ampicillin and oxacillin, in four bacterial pathogens [14]. Two 6-oxophenolic triterpenoids, zeylasteral and demethylzeylasteral, which were isolated from the root of *Maytenus blepharodes*, have antimicrobial activity against *Bacillus subtilis* [35]. Those triterpenoids block cell division by inhibiting DNA synthesis and macromolecular synthesis in *Bacillus subtilis*. In addition, sesquiterpene farnesol can inhibit recycling of the lipid carrier of the murein monomer precursor and also reduce the secretion and activity of β-lactamase, thus contributing to increased susceptibility to β-lactams in methicillin-resistant *S. aureus* [36]. In this study, we found that both oleanolic acid and ursolic acid have antibacterial activity against Gram-positive bacteria, especially *E. faecalis*, *L. monocytogenes*, *B. cereus* and *S. aureus*. Our data suggest that triterpenoids in combination with ampicillin or tetracycline may lead to a synergistic effect theoretically. Their ability to enhance the activity of β-lactams could constitute a valuable group of therapeutic agents in the future.

## 4. Materials and Methods

### 4.1. Plant Materials

The leaves of *Alstonia scholaris* (L.) R. Br. were collected from the wild field in March, June, September, and December of 2011 and 2012. The field site is located in an *A. scholaris* forest near Mingdao University (N 23°52′15.17’’ and E 120°29’47.13’’), Changhua County, Taiwan. The plant species was identified by Dr. Tsai-Wen Hsu, key laboratory of High Altitude Experimental Station, Taiwan Endemic Species Research Institute. The voucher specimens (2010-0118-Jhan) were deposited in the Chemical Ecology Lab., Research Center for Biodiversity, China Medical University.

### 4.2. Bioassay Guided Chromatography

All fractions were dissolved in DMSO (Sigma, St. Louis, MO, USA) to reach a final concentration of 50 μg/μL. Sterile discs (6 mm), filled with 10 μL of each fraction, were placed on Muller-Hinton agar (MH agar). The bacterial inoculums were prepared from overnight broth culture in normal saline (0.85% of NaCl) in order to obtain an optical density ranging from 0.08 to 0.1 at 590 nm. DMSO was used as negative control. Ampicillin for bacteria was used as positive control. Bacterial growth inhibition was determined as the diameter of the inhibition zones around the discs. Diameters (mm) of the inhibition zone were measured and documented after incubation at 37 °C for 18–24 h.

### 4.3. Isolation and Identification of Triterpenoids

Five kilograms of air-dried leaves of *A. scholaris* were extracted with methanol thrice followed the standard extraction procedures [37]. The methanolic extract was concentrated to gain 395 g dry residue and then partitioned by hexane, ethyl acetate, butanol with H_2_O to the obtained portion of *n*-hexane (80.59 g), EtOAc (131.15 g), BuOH (37.59 g), and an aqueous layer (45.44 g). Bioassay guided chromatography was carried out for antibacterial compound isolation (Table 1). The EtOAc portion was subjected to a silica gel column in gradient elution of mixture solvent composed of hexane-ethyl acetate and led to 34 fractions (EA-1~EA-34). Fractions with antibacterial activities were further subjected to column chromatography for potent chemical constitute isolation. Fraction EA-8 (17.2 g) was separated via silica gel column with a hexane-ethyl acetate mixture as the eluent to obtain 23 subfractions (EA-8-1~EA-8-23) and compound **1** (5.3 mg) was purified from subfraction EA-8-4. Fraction EA-8-12 was further fractionated by another silica gel column with a hexane-ethyl acetate mixture (10:1) to give 21 subfractions (EA-8-12-1~EA-8-12-21). Compounds **2** (32.4 mg), **3** (23.5 mg) and **4** (12.6 mg) were isolated form fractions 8-12-4, 8-12-5 by reverse phase C-18 chromatography in gradient elution of MeOH–H_2_O (60% to 100%). In addition, Compound **5** (40.7 mg) and **6** (102.2 mg) were isolated from the effective fraction of EtOAc portion, fraction EA-12 (3.2 g), by reverse phase C-18 chromatography in gradient elution of MeOH–H_2_O (70% to 100%). Purified compounds were subjected to spectroscopic identification by using ^1^H-NMR and ^13^C-NMR (Bruker Avance 400) and Mass (Bruker Daltonics Esquire HCT). The isolated compounds were identified by comparison of spectra data with literature reported previously. Detail structure elucidation of six triterpenoids isolated from *A. scholaris* in this study was decribed in Supplementary Materials.

*Lupeol* (**1**): White crystal; ESI-MS *m/z* 449.6 [M + Na]^+^ (Calcd for C_30_H_50_O: 426.3); ^1^H-NMR spectrum (400 MHz, CDCl_3_): δ 4.68 (1H, brs, Hβ-29), 4.56 (1H, brs, Hα-29), 3.16 (1H, dd, *J* = 11.2, 4.8 Hz, H-3), 2.36 (1H, dt, *J* = 12.9, 6.0 Hz, H-19), 1.68 (3H, s, Me-30), 1.03, 0.97, 0.95, 0.83, 0.79, 0.76 (Me-26, Me-23, Me-27, Me-25, Me-28, Me-24), 0.67 (1H, d, *J* = 9.2 Hz, H-5). ^13^C-NMR spectrum (100 MHz, CDCl_3_): δ C: 38.7 (C-1), 27.4 (C-2), 79.0 (C-3), 38.8 (C-4), 55.3 (C-5), 18.3 (C-6), 34.2 (C-7), 40.8 (C-8), 50.4 (C-9), 37.1 (C-10), 20.9 (C-11), 25.1 (C-12), 38.0 (C-13), 42.8 (C-14), 27.4 (C-15), 35.6 (C-16), 43.0 (C-17), 48.0 (C-18), 48.3 (C-19), 150.9 (C-20), 29.8 (C-21), 40.0 (C-22), 28.0 (C-23), 15.3 (C-24), 15.9 (C-25), 16.1 (C-26), 14.5 (C-27), 18.0 (C-28), 109.5 (C-29), 19.3 (C-30).

*Betulin* (**2**): White amorphous powder; ESI-MS *m/z* 465.6 [M + Na]^+^ (Calcd for C_30_H_50_O_2_: 442.3); ^1^H-NMR spectrum (600 MHz, CDCl_3_): δ 4.68 (1H, brs, Hβ-29), 4.58 (1H, brs, Hα-29), 3.18 (1H, dd, *J* = 11.4, 4.8 Hz, H-3), 2.37 (1H, m, H-19), 1.68 (3H, s, Me-30), 1.02, 0.97, 0.96, 0.82, 0.76 (Me-26, Me-27, Me-23, Me-25, Me-24), 0.67 (1H, d, *J* = 9.0 Hz, H-5). ^13^C-NMR spectrum (100 MHz, CDCl_3_): δ C: 38.6 (C-1), 27.3 (C-2), 78.9 (C-3), 38.8 (C-4), 55.2 (C-5), 18.2 (C-6), 34.1 (C-7), 40.8 (C-8), 50.3 (C-9), 37.1 (C-10), 20.7 (C-11), 25.1 (C-12), 37.2 (C-13), 42.6 (C-14), 26.9 (C-15), 29.1 (C-16), 47.7 (C-17), 48.7 (C-18), 47.6 (C-19), 150.4 (C-20), 29.6 (C-21), 34.1 (C-22), 27.9 (C-23), 15.3 (C-24), 16.0 (C-25), 15.9 (C-26), 14.7 (C-27), 60.5 (C-28), 109.6 (C-29), 19.0 (C-30).

*3-Hydroxy-11-ursen-28,13-olide* (**3**): White amorphous powder; ESI-MS *m/z* 477.3 [M + Na]^+^ (Calcd for C_30_H_46_O_3_: 454.3); ^1^H-NMR spectrum (400 MHz, pyridine-*d*_5_): δ 5.98 (1H, d, *J* = 9.6 Hz, H-11), 5.62 (1H, d, *J* = 9.6 Hz, H-12), 3.41 (1H, dd, H-3), 1.19 (3H, s, Me-27), 1.17 (3H, s, Me-23), 1.15 (3H, s, Me-25), 0.96 (3H, s, Me-26), 0.87 (3H, s, Me-29), 0.82 (3H, s, Me-30), 0.75 (3H, s, Me-24). ^13^C-NMR spectrum (100 MHz, pyridine-*d*_5_): δ C: 36.5 (C-1), 25.8 (C-2), 77.9 (C-3), 39.4 (C-4), 55.0 (C-5), 19.2 (C-6), 31.9 (C-7), 41.9 (C-8), 53.3 (C-9), 38.5 (C-10), 133.6 (C-11), 129.3 (C-12), 89.5 (C-13), 42.1 (C-14), 27.8 (C-15), 23.1 (C-16), 45.1 (C-17), 60.4 (C-18), 38.1 (C-19), 40.2 (C-20), 30.9 (C-21), 31.4 (C-22), 28.3 (C-23), 16.1 (C-24), 19.2 (C-25), 19.1 (C-26), 15.8 (C-27), 179.3 (C-28), 17.8 (C-29), 18.1 (C-30).

*Betulinic acid* (**4**): White amorphous powder; ESI-MS *m/z* 455.3 [M − H]^−^ (Calcd for C_30_H_48_O_3_: 456.3); ^1^H-NMR spectrum (400 MHz, pyridine-*d*_5_): δ 4.91 (1H, brs, Hβ-29), 4.74 (1H, brs, Hα-29), 3.53 (1H, m, H-19), 3.40 (1H, t, *J* = 8.0 Hz, H-3), 2.68 (1H, m, H-13), 2.58 (1H, d, *J* = 12.0, H-16), 2.21 (1H, m, H-21), 1.77 (3H, s, Me-30), 1.19, 1.05, 1.03, 0.98, 0.80 (Me-23, Me-27, Me-26, Me-24, Me-25). ^13^C-NMR (100 MHz, pyridine-*d*_5_): δ C: 39.2 (C-1); 27.9 (C-2); 77.8 (C-3); 39.2 (C-4); 55.6 (C-5); 18.4 (C-6); 34.5 (C-7); 40.8 (C-8); 49.4 (C-9); 37.2 (C-10); 20.9 (C-11); 25.8 (C-12); 38.3 (C-13); 42.5 (C-14); 29.9 (C-15); 32.5 (C-16); 56.3 (C-17); 47.4 (C-18); 49.4 (C-19); 151.0 (C-20); 30.9 (C-21); 37.2 (C-22); 28.3 (C-23); 16.1 (C-24); 16.0 (C-25); 19.1 (C-26); 14.6 (C-27); 178.5 (C-28); 109.6 (C-29); 19.1 (C-30).

*Oleanolic acid* (**5**): White amorphous powder; ESI-MS *m/z* 479.3 [M + Na]^+^ (Calcd for C_30_H_48_O_3_: 456.3); ^1^H-NMR spectrum (400 MHz, pyridine-*d*_5_):δ 5.47 (1H, s, H-12), 3.42 (1H, dd, *J* = 5.6, 9.6 Hz, H-3), 3.31 (1H, dd, *J* = 10.4, H-18), 1.26 (3H, s, Me-27), 1.22, 1.03, 1.03, 1.00, 0.99, 0.93 (Me-23, Me-26, Me-30, Me-24, Me-29, Me-25). ^13^C-NMR (100 MHz, pyridine-*d*_5_): δ C: 38.8 (C-1); 28.0 (C-2); 78.0 (C-3); 39.3 (C-4); 55.7 (C-5); 18.7 (C-6); 33.2 (C-7); 39.6 (C-8); 48.0 (C-9); 37.3 (C-10); 23.6 (C-11); 122.4 (C-12); 144.8 (C-13); 42.1 (C-14); 28.2 (C-15); 23.7 (C-16); 46.6 (C-17); 41.9 (C-18); 46.4 (C-19); 30.9 (C-20); 34.2 (C-21); 33.2 (C-22); 28.7 (C-23); 16.5 (C-24); 15.6 (C-25); 17.5 (C-26); 26.1 (C-27); 180.4 (C-28); 33.2 (C-29); 23.7 (C-30).

*Ursolic acid* (**6**): White amorphous powder; ESI-MS *m/z* 479.3 [M + Na]^+^ (Calcd for C_30_H_48_O_3_: 456.3); ^1^H-NMR spectrum (400 MHz, pyridine-*d*_5_):δ 5.46 (1H, s, H-12), 3.43 (1H, dd, *J* = 6.8, 8.8 Hz, H-3), 2.62 (1H, d, *J* = 11.2, H-18), 1.22 (3H, s, Me-23), 1.21, 1.02, 1.02, 1.00, 0.97, 0.93 (Me-27, Me-26, Me-29, Me-24, Me-30, Me-25). ^13^C-NMR (100 MHz, pyridine-*d*_5_): δ C: 39.0 (C-1); 28.0 (C-2); 78.0 (C-3); 39.4 (C-4); 55.7 (C-5); 18.7 (C-6); 33.5 (C-7); 39.9 (C-8); 48.0 (C-9); 37.4 (C-10); 23.5 (C-11); 125.5 (C-12); 139.2 (C-13); 42.4 (C-14); 28.6 (C-15); 24.8 (C-16); 48.0 (C-17); 53.5 (C-18); 39.3 (C-19); 39.2 (C-20); 31.0 (C-21); 37.2 (C-22); 28.7 (C-23); 16.5 (C-24); 15.6 (C-25); 17.4 (C-26); 23.8 (C-27); 179.9 (C-28); 17.4 (C-29); 21.3 (C-30).

### 4.4. Bacterial Strains and Media

Eight pathogenic standard strains obtained from American Type Culture Collection (ATCC) (Manassas, VA, USA) are *Bacillus cereus* (ATCC 9139), *Enterococcus faecalis* (ATCC 29212), *Escherichia coli* (ATCC 35150), *Listeria monocytogenes* (ATCC 7644), *Pseudomonas aeruginosa* (ATCC 27853), *Salmonella enterica* (ATCC 13311), methicillin-sensitive *Staphylococcus aureus* (MSSA, ATCC 29213), and methicillin-resistant *Staphylococcus aureus* (MRSA, ATCC 43300), which were employed to evaluate the antibacterial assay. All bacterial strains were cultured on nutrient agar (Difco, Sparks, MD, USA) or in nutrient broth (Difco).

### 4.5. Minimal Inhibitory Concentration (MIC) Determination

MICs were determined by the broth micro-dilution method according to the guidelines of the Clinical and Laboratory Standards Institute [38]. Detailed experimental processes were established according to previous reports [39]. Briefly, all bacteria strains were cultured on nutrient agar (Difco) and incubated at 37 °C for 24 h. Bacterial inoculums were prepared in normal saline and diluted to give a final density of 5 × 10^5^ cfu/mL by comparison with a 0.5 McFarland turbidity standard. All compounds were dissolved in DMSO (Sigma) and then in nutrient broth to reach a final concentration of 256 μg/mL. Serial two-fold dilutions were made in a concentration range from 0.25 to 128 μg/mL. In each microtiter plate (Corning, Lowell, MA, USA), all of the wells were examined by spectrophotometer at 600 nm for bacterial growth. The MIC was defined as the lowest concentration at which no visible growth occurred in comparison with positive control. A column with the antibiotics (ampicillin and tetracycline) was used as a positive control. At least three independent determinations were repeated.

### 4.6. Synergistic Effects of Drug Combination

The antagonistic, indifferent or synergistic effect of triterpenoids in combination with antibiotics was determined by using the FICI.The growth medium was supplemented with various concentrations of compounds, ranging from 1/64 to 4 folds of MIC. The instruction of checkerboard assay reported previously [40] was described as the following. One plate was used to make antibacterial dilutions of antibiotics in the vertical orientation. Another one was used to make serial two-fold dilutions of triterpenoids in horizontal direction. Both dilutions were prepared in nutrient broth (50 μL per well). The plate of antibiotic was then transferred to plates with triterpenoids and 100 µL of bacterial suspension was added to each well to give a final density of 5 × 10^5^ cfu/mL. The concentrations of antibiotics and triterpenoids were selected on the basis of the MIC values described previously. For testing the activity of antibiotics and triterpenoids, two formulas, FIC_A_ and FIC_B_, were employed. FIC_A_ is the MIC of triterpenoid in the presence of antibiotic/MIC of triterpenoid alone, and FIC_B_ is the MIC of an antibiotic in the presence of triterpenoid/MIC of antibiotic alone. The formulas are given as follows:

$${\mathrm{FIC}}_{\text{A}}=\frac{\text{MIC of triterpenoid in the present of antibiotic}}{\text{MIC of triterpenoid alone}}$$

$${\mathrm{FIC}}_{\text{B}}=\frac{\text{MIC of antibiotic in the present of triterpenoid}}{\text{MIC of antibiotic alone}}$$

FICI is the sum of FIC_A_ and FIC_B_. The meaning of FICI is represented as synergistic effect (≤0.5), indifference effect (0.5~4) and antagonism effect (≥4) [18].

### 4.7. Time-Kill Assay

The overnight cultures of *S. aureus* and *B. cereus* were prepared in nutrient broth and diluted to give a final density of 1.5 × 10^5^ cfu/mL by comparison with a 0.5 McFarland turbidity standard. The bacteria were incubated with 1/2 MIC of triterpenoids and ampicillin alone or in combination. After 2, 4, 6, 18 and 24 h incubation, 100 μL of bacterial aliquots were taken and spread onto nutrient agar for colony-forming unit (CFU) counting. A reduction of >2 log_10_ in the cell count obtained in the present of triterpenoids and antibiotics was interpreted as synergy [19].

## 5. Conclusions

Six pentacyclic triterpenoids have been isolated from leaves of *A. scholaris*. Both oleanolic acid and ursolic acid showed antibacterial activity but were limited to Gram-positive bacteria. Ursolic acid showed a synergistic effect with ampicillin and tetracycline against both *Bacillus cereus* and *S. aureus*, respectively. The ability of pentacyclic triterpenoid to enhance the activity of β-lactams could constitute a valuable group of therapeutic agents in the future.

## Supplementary Materials

Supplementary materials can be accessed at: http://www.mdpi.com/1420-3049/21/2/139/s1.

## References

[1] Baliga M.S. (2012). Review of the phytochemical, pharmacological and toxicological properties of *Alstonia scholaris* Linn. R. Br (Saptaparna). Chin. J. Integr. Med..

[2] Shang J.H., Cai X.H., Zhao Y.L., Feng T., Luo X.D. (2010). Pharmacological evaluation of *Alstonia scholaris*: Anti-tussive, anti-asthmatic and expectorant activities. J. Ethnopharmacol..

[3] Khyade M.S., Kasote D.M., Vaikos N.P. (2014). *Alstonia scholaris* (L.) R. Br. and *Alstonia macrophylla* Wall. ex G. Don: A comparative review on traditional uses, phytochemistry and pharmacology. J. Ethnopharmacol..

[4] El-Askary H.I., El-Olemy M.M., Salama M.M., Sleem A.A., Amer M.H. (2012). Bioguided isolation of pentacyclic triterpenes from the leaves of *Alstonia scholaris* (Linn.) R. Br. growing in Egypt. Nat. Prod. Res..

[5] Wang F., Ren F.C., Liu J.K. (2009). Alstonic acids A and B, unusual 2,3-secofernane triterpenoids from *Alstonia scholaris*. Phytochemistry.

[6] Cai X.H., Tan Q.G., Liu Y.P., Feng T., Du Z.Z., Li W.Q., Luo X.D. (2008). A cage-monoterpene indole alkaloid from *Alstonia scholaris*. Org. Lett..

[7] Gandhi M., Vinayak V.K. (1990). Preliminary evaluation of extracts of *Alstonia scholaris* bark for *in vivo* antimalarial activity in mice. J. Ethnopharmacol..

[8] Lin S.C., Lin C.C., Lin Y.H., Supriyatna S., Pan S.L. (1996). The protective effect of *Alstonia scholaris* R. Br. on hepatotoxin-induced acute liver damage. Am. J. Chin. Med..

[9] Jagetia G.C., Baliga M.S. (2006). Evaluation of anticancer activity of the alkaloid fraction of *Alstonia scholaris* (Sapthaparna) *in vitro* and *in vivo*. Phytother. Res..

[10] Shang J.H., Cai X.H., Feng T., Zhao Y.L., Wang J.K., Zhang L.Y., Yan M., Luo X.D. (2010). Pharmacological evaluation of *Alstonia scholaris*: Anti-inflammatory and analgesic effects. J. Ethnopharmacol..

[11] Saraswathi V., Ramamoorthy N., Subramaniam S., Mathuram V., Gunasekaran P., Govindasamy S. (1998). Inhibition of glycolysis and respiration of sarcoma-180 cells by echitamine chloride. Chemotherapy.

[12] Bonvicini F., Mandrone M., Antognoni F., Poli F., Gentilomi G.A. (2014). Ethanolic extracts of *Tinospora cordifolia* and *Alstonia scholaris* show antimicrobial activity towards clinical isolates of methicillin-resistant and carbapenemase-producing bacteria. Nat. Prod. Res..

[13] Cushnie T.P., Lamb A.J. (2011). Recent advances in understanding the antibacterial properties of flavonoids. Int. J. Antimicrob. Agents.

[14] Kurek A., Nadkowska P., Pliszka S., Wolska K.I. (2012). Modulation of antibiotic resistance in bacterial pathogens by oleanolic acid and ursolic acid. Phytomedicine.

[15] Wagner H., Ulrich-Merzenich G. (2009). Synergy research: Approaching a new generation of phytopharmaceuticals. Phytomedicine.

[16] Walencka E., Rozalska S., Wysokinska H., Rozalski M., Kuzma L., Rozalska B. (2007). Salvipisone and aethiopinone from *Salvia sclarea* hairy roots modulate staphylococcal antibiotic resistance and express anti-biofilm activity. Planta Med..

[17] Brehm-Stecher B.F., Johnson E.A. (2003). Sensitization of *Staphylococcus aureus* and *Escherichia coli* to antibiotics by the sesquiterpenoids nerolidol, farnesol, bisabolol, and apritone. Antimicrob. Agents Chemother..

[18] Odds F.C. (2003). Synergy, antagonism, and what the chequerboard puts between them. J. Antimicrob. Chemother..

[19] European Committee for Antimicrobial Susceptibility Testing (EUCAST) of the European Society of Clinical Microbiology and Infectious Dieases (ESCMID) (2000). Teriminology relating to methods for determination of susceptibility of bacteria to antimicrobial agents. Clin. Microbiol. Infec..

[20] Moellering R.C. (2012). MRSA: The first half century. J. Antimicrob. Chemother..

[21] Stefani S., Chung D.R., Lindsay J.A., Friedrich A.W., Kearns A.M., Westh H., Mackenzie F.M. (2012). Meticillin-resistant *Staphylococcus aureus* (MRSA): Global epidemiology and harmonisation of typing methods. Int. J. Antimicrob. Agents.

[22] Bottone E.J. (2010). *Bacillus cereus*, a volatile human pathogen. Clin. Microbiol. Rev..

[23] Kiyomizu K., Yagi T., Yoshida H., Minami R., Tanimura A., Karasuno T., Hiraoka A. (2008). Fulminant septicemia of *Bacillus cereus* resistant to carbapenem in a patient with biphenotypic acute leukemia. J. Infect. Chemother..

[24] Kudaka J., Horikawa K., Uryu K., Matsuyuki S., Ogata K., Kawano K., Yamaguchi Y., Yamasaki S., Watanabe H., Iwanaga M. (2005). Symptoms of food-borne diseases and gastroenteritis in Kyushu, Japan. Kansenshogaku Zasshi.

[25] Chang J.M., Chen T.H. (2003). Bacterial foodborne outbreaks in central Taiwan, 1991–2000. J. Food Drug Anal..

[26] Cho J.I., Lee S.H., Lim J.S., Koh Y.J., Kwak H.S., Hwang I.G. (2011). Detection and distribution of food-borne bacteria in ready-to-eat foods in Korea. Food Sci. Biotechnol..

[27] Hall J.A., Goulding J.S., Bean N.H., Tauxe R.V., Hedberg C.W. (2001). Epidemiologic profiling: Evaluating foodborne outbreaks for which no pathogen was isolated by routine laboratory testing: United States, 1982–1989. Epidemiol. Infect..

[28] Cowan M.M. (1999). Plant products as antimicrobial agents. Clin. Microbiol. Rev..

[29] Tsutsumi L.S., Owusu Y.B., Hurdle J.G., Sun D.Q. (2014). Progress in the discovery of treatments for *C. difficile* Infection: A clinical and medicinal chemistry review. Curr. Top. Med. Chem..

[30] Ito J., Chang F.R., Wang H.K., Park Y.K., Ikegaki M., Kilgore N., Lee K.H. (2001). Anti-AIDS agents. 48.(1) Anti-HIV activity of moronic acid derivatives and the new melliferone-related triterpenoid isolated from Brazilian propolis. J. Nat. Prod..

[31] Kurek A., Grudniak A.M., Szwed M., Klicka A., Samluk L., Wolska K.I., Janiszowska W., Popowska M. (2010). Oleanolic acid and ursolic acid affect peptidoglycan metabolism in *Listeria monocytogenes*. Antonie van Leeuwenhoek.

[32] Wang C.M., Chen H.T., Li T.C., Weng J.H., Jhan Y.L., Lin S.X., Chou C.H. (2014). The role of pentacyclic triterpenoids in the allelopathic effects of *Alstonia scholaris*. J. Chem. Ecol..

[33] Way T.D., Tsai S.J., Wang C.M., Ho C.T., Chou C.H. (2014). Chemical constituents of *Rhododendron formosanum* show pronounced growth inhibitory effect on non-small-cell lung carcinoma cells. J. Agric. Food Chem..

[34] Frere J.M. (1977). Mechanism of action of beta-lactam antibiotics at the molecular level. Biochem. Pharmacol..

[35] De Leon L., Beltran B., Moujir L. (2005). Antimicrobial activity of 6-oxophenolic triterpenoids. Mode of action against *Bacillus subtilis*. Planta Med..

[36] Kuroda M., Nagasaki S., Ohta T. (2007). Sesquiterpene farnesol inhibits recycling of the C55 lipid carrier of the murein monomer precursor contributing to increased susceptibility to beta-lactams in methicillin-resistant *Staphylococcus aureus*. J. Antimicrob. Chemother..

[37] Wang C.M., Jhan Y.L., Yen L.S., Su Y.H., Chang C.C., Wu Y.Y., Chang C.I., Tsai S.Y., Chou C.H. (2013). The allelochemicals of litchi leaf and its potential as natural herbicide in weed control. Allelopath. J..

[38] Clinical and Laboratory Standards Institute (CLSI) (2015). Methods for Dilution Antimicrobial Susceptibility Tests for Bacteria That Grow Aerobically.

[39] Wang C.M., Hsu Y.M., Jhan Y.L., Tsai S.J., Lin S.X., Su C.H., Chou C.H. (2015). Structure elucidation of procyanidins isolated from *Rhododendron formosanum* and their anti-oxidative and anti-bacterial activities. Molecules.

[40] Hemaiswarya S., Doble M. (2009). Synergistic interaction of eugenol with antibiotics against Gram negative bacteria. Phytomedicine.

