# Supplementary material for: Antibacterial and Synergistic Activity of Pentacyclic Triterpenoids Isolated from Alstonia scholaris
Source: Molecules. 2016 Jan 25;21(2):139. doi: 10.3390/molecules21020139 (PMC6273878; doi:10.3390/molecules21020139)
Supplement: Supplementary file 1 [file molecules-21-00139-s001.pdf]

Supplementary Materials: Antibacterial and Synergistic Activity of Pentacyclic Triterpenoids Isolated from *Alstonia scholaris*

Chao-Min Wang [1], Hsiao-Ting Chen [1], Zong-Yen Wu [2], Yun-Lian Jhan [1], Ching-Lin Shyu [2] and Chang-Hung Chou [1,*]

Structural elucidation of compound **1**–**6**

The ESI-MS of compound **1** recorded in negative-ion modes exhibited a deprotonated ion $[M + Na]^+$ at *m/z* 449.6, indicating molecular formulas of $C_{30}H_{50}O$. The ^{1}H-NMR spectrum ($CDCl_3$, 600 MHz) exhibited seven signals as singlet to δ 0.97 (3H, s, Me-23), 0.76 (3H, s, Me-24), 0.83 (3H, s, Me-25), 1.03 (3H, s, Me-26), 0.95 (3H, s, Me-27), 0.79 (3H, s, Me-28) and at lower fields other to δ 1.68 (3H, s, Me-30) assignable to a methyl group on double bond; a multiplet at δ 1.94 (2H, m, H-21); a double doublet to δ 2.36 (1H, dd, *J* = 12.9 and 6.0 Hz, H-19) to be assigned to a methynic proton; a double doublet to δ 3.16 (1H, dd, *J* = 11.2 and 4.8 Hz, H-3) assignable to a terminal proton of a secondary alcohol, two doublets for proton to an exocyclic methylene to δ 4.55 (2H, d, *J* = 2.0 Hz, H-29) and 4.67 (2H, d, *J* = 2.0 Hz, H-29). ^{13}C-NMR ($CDCl_3$, 150 MHz) showed 30 carbon signals; vinyl carbon signal occurs at 109.3 ppm, the signal corresponding to methylene-methylidene at 150.9 ppm and the carbon bound to the hydroxyl group appears at 79.0 ppm. Comparison of the ^{1}H- and ^{13}C-NMR spectroscopic data with the literature [1] established compound **1** as lupeol.

The mass spectrum suggested formula $C_{30}H_{50}O_2$ ($[M + Na]^+$, *m/z* 465.6) of compound **2**. Its ^{1}H-NMR spectrum ($CDCl_3$, 600 MHz) exhibited six signals as singlet to δ 1.02 (3H, s, Me-26), 0.75 (3H, s, Me-24), 0.82 (3H, s, Me-25), 0.96 (3H, s, Me-23), 0.97 (3H, s, Me-27), 1.68 (3H, s, Me-30); a multiplet at δ 2.37 (2H, m, H-19); a double doublet to δ 3.18 (1H, dd, *J* = 11.4 and 4.8 Hz, H-3) assignable to a terminal proton of a secondary alcohol, two doublets for proton to an exocyclic methylene to δ 4.55 (2H, d, *J* = 2.0 Hz, H-29) and 4.67 (2H, d, *J* = 2.0 Hz, H-29). 30 carbon signals were observed in ^{13}C-NMR ($CDCl_3$, 150 MHz) spectrum; there was vinyl carbon signal at 109.6 ppm, the signal corresponding to methylene-methylidene at 150.4 ppm and the carbon bound to the hydroxyl group appears at 78.9 ppm. Comparison of the ^{1}H- and ^{13}C-NMR spectroscopic data with the literature [1] established compound **2** as betulin.

The ESI-MS spectrum suggested formula $C_{30}H_{46}O_3$ ($[M + Na]^+$, *m/z* 477.3) of compound **3**. Its ^{1}H-NMR spectrum ($CDCl_3$, 400 MHz), exhibited seven signals as singlet to 1.17 (3H, s, H-23), 0.75 (3H, s, H-24), 1.15 (3H, s, H-25), 0.96 (3H, s, H-26), 1.19 (3H, s, H-27), 0.87 (3H, d, *J* = 6.0 Hz, H-29), 0.82 (3H, d, *J* = 6.0 Hz, H-30); two signals of olefinic proton at δ 5.62, 5.98 (H, dd, H-12, H-11); and a carbinolic proton at δ 3.41 (dd, H-3), referring to the axial and α orientation. The ^{13}C-NMR spectrum showed the presence of 30 carbons comprising 7 methyls (δ 15.8, 16.1, 17.8, 18.1, 19.1, 19.2, 28.3), 9 methylenes (δ 19.2, 23.1, 25.8, 27.8, 30.9, 31.4, 31.9, 36.5, 38.1), 4 methines (δ60.4, δ77.9, δ129.3, δ 133.6), and 8 quaternary carbons (δ 38.5, 39.4, 40.2, 41.9, 42.1, 45.1, 89.3, δ 179.3). On the basis of the above observations, the NMR spectral data of compound **3** were similar to those previously reported for 3-hydroxy-11-oleane-28,13-olide [2].

The mass spectrum suggested formula $C_{30}H_{48}O_3$ ($[M - H]^-$, *m/z* 455.3) of compound **4**. The ^{1}H-NMR spectrum revealed the presence of 6 singlet methyls at δ 1.19 (3H, s, Me-23), 0.98 (3H, s, Me-24), 0.80 (3H, s, Me-25), 1.03 (3H, s, Me-26), 1.05 (3H, s, Me-27), and 1.77 (3H, s, Me-30); a pair of olefinic protons at δ 4.91 and δ 4.74 (each one H, s), characteristic of an exocyclic methylene group; and a carbinolic proton at δ 3.40 (t, *J* = 8.0 Hz, H-3), referring to the axial and α orientation. Further, δ 3.53 (m, H-19), δ 2.58 (d, *J* = 12.0 Hz, H-16), δ 2.68 (m, H-13), and δ 2.21 (m, H-21) were characteristic of a lupine triterpenoid [3]. The ^{13}C-NMR spectrum showed the presence of 30 carbons comprising 6 methyls (δ 14.6, 16.0, 16.1, 19.1, 19.1, 28.3), 11 methylenes (δ 18.4, 20.9, 25.8, 27.9, 29.9, 30.9, 32.5, 34.5, 37.2, 39.2, 109.6), 6 methines (δ 38.3, 47.4, 49.4, 49.4, 55.6, 77.8), and 7 quaternary carbons (δ 37.2, 39.2, 40.8,

42.5, 56.3, 151.0, 178.5). On the basis of the above analyses, the NMR spectral data of compound **4** were similar to those previously reported for betulinic acid [3].

On the basis of positive ESI-MS of the molecular ion peak at *m/z* 479.40 ([M + Na]$^+$), matched with the number of carbons and hydrogens recorded by using NMR spectroscopy, we identified Compound **5** with the molecular formula $C_{30}H_{48}O_3$ (calculated, 456.36). The ^{1}H-NMR spectrum revealed the presence of 7 singlet methyls at δ 1.22 (3H, s, Me-23), 1.0 (3H, s, Me-24), 0.93 (3H, s, Me-25), 1.03 (3H, s, Me-26), 1.26 (3H, s, Me-27), 0.99 (3H, s, Me-29), and 1.03 (3H, s, Me-30); a signal of olefinic proton at δ 5.47 (H, s, H-12); and a carbinolic proton at δ 3.42 (dd, *J* = 5.6, 9.6 Hz, H-3), referring to the characteristic axial and *α* orientation of oleanolic triterpenoid. The ^{13}C-NMR spectra showed the presence of 30 carbons comprising 7 methyls (δ 15.6, 16.5, 17.5, 23.7, 26.1, 28.7, 33.2), 10 methylenes (δ 18.7, 23.6, 23.7, 28.0, 28.2, 33.2, 33.2, 34.2, 38.8, 46.4), 5 methines (δ 41.9, 48.0, 55.7, 78.0, 122.4), and 8 quaternary carbons (δ 30.9, 37.3, 39.3, 39.6, 42.1, 46.6, 144.8, 180.4). On the basis of the above observations, the NMR spectral data of compound **5** were similar to those previously reported for oleanolic acid [4].

The ^{1}H- and ^{13}C-NMR data were consistent with those previously reported [4]. On the basis of positive ESI-MS of the molecular ion peak at *m/z* 479.40 ([M + Na]$^+$), matched with the number of carbons and hydrogens recorded by using NMR spectroscopy, we obtained Compound **6** with the molecular formula $C_{30}H_{48}O_3$ (calculated, 456.3). On the basis of ^{13}C-NMR and DEPT, we determined the presence of 5 rings, plus 1 acidic carbonyl group and 1 olefinic group, in the molecule. The ^{1}H-NMR spectrum revealed the presence of 7 singlet methyls at δ_H 1.22 (3H, s, Me-23), 1.00 (3H, s, Me-24), 0.93 (3H, s, Me-25), 1.02 (3H, s, Me-26), 1.21 (3H, s, Me-27), 1.02 (3H, s, Me-29), and 0.97 (3H, s, Me-30); a signal of olefinic proton at δ 5.46 (H, s, H-12); and a carbinolic proton at δ 3.43 (dd, *J* = 6.8, 8.8 Hz, H-3), referring to the axial and *α* orientation. The ^{13}C-NMR spectrum showed the presence of 30 carbons comprising 7 methyls (δ 15.6, 16.5, 17.4, 17.4, 21.3, 23.8, 28.7), 9 methylenes (δ 18.7, 23.5, 24.8, 28.0, 28.6, 31.0, 33.5, 37.2, 39.0), 7 methines (δ 39.2, 39.3, 48.0, 53.5, 55.7, 78.0, 125.5), and 7 quaternary carbons (δ 37.4, 39.4, 39.9, 42.4, 48.0, 139.2, 179.9). On the basis of the above observations, the NMR spectral data of compound **6** were similar to those previously reported for ursolic acid [4].

Lupeol (**1**): White crystal; ESI-MS *m/z* 449.6 [M + Na]$^+$ (Calcd for $C_{30}H_{50}O$: 426.3); ^{1}H-NMR spectrum (400 MHz, $CDCl_3$): δ 4.68 (1H, brs, Hβ-29), 4.56 (1H, brs, H*α*-29), 3.16 (1H, dd, *J* = 11.2, 4.8 Hz, H-3), 2.36 (1H, dt, *J* = 12.9, 6.0 Hz, H-19), 1.68 (3H, s, Me-30), 1.03, 0.97, 0.95, 0.83, 0.79, 0.76 (Me-26, Me-23, Me-27, Me-25, Me-28, Me-24), 0.67 (1H, d, *J* = 9.2 Hz, H-5). ^{13}C-NMR spectrum (100 MHz, $CDCl_3$): δ C: 38.7 (C-1), 27.4 (C-2), 79.0 (C-3), 38.8 (C-4), 55.3 (C-5), 18.3 (C-6), 34.2 (C-7), 40.8 (C-8), 50.4 (C-9), 37.1 (C-10), 20.9 (C-11), 25.1 (C-12), 38.0 (C-13), 42.8 (C-14), 27.4 (C-15), 35.6 (C-16), 43.0 (C-17), 48.0 (C-18), 48.3 (C-19), 150.9 (C-20), 29.8 (C-21), 40.0 (C-22), 28.0 (C-23), 15.3 (C-24), 15.9 (C-25), 16.1 (C-26), 14.5 (C-27), 18.0 (C-28), 109.5 (C-29), 19.3 (C-30).

Betulin (**2**): white amorphous powder; ESI-MS *m/z* 465.6 [M + Na]$^+$ (Calcd for $C_{30}H_{50}O_2$: 442.3); ^{1}H-NMR spectrum (600 MHz, $CDCl_3$): δ 4.68 (1H, brs, Hβ-29), 4.58 (1H, brs, H*α*-29), 3.18 (1H, dd, *J* = 11.4, 4.8 Hz, H-3), 2.37 (1H, m, H-19), 1.68 (3H, s, Me-30), 1.02, 0.97, 0.96, 0.82, 0.76 (Me-26, Me-27, Me-23, Me-25, Me-24), 0.67 (1H, d, *J* = 9.0 Hz, H-5). ^{13}C-NMR spectrum (100 MHz, $CDCl_3$): δ C: 38.6 (C-1), 27.3 (C-2), 78.9 (C-3), 38.8 (C-4), 55.2 (C-5), 18.2 (C-6), 34.1 (C-7), 40.8 (C-8), 50.3 (C-9), 37.1 (C-10), 20.7 (C-11), 25.1 (C-12), 37.2 (C-13), 42.6 (C-14), 26.9 (C-15), 29.1 (C-16), 47.7 (C-17), 48.7 (C-18), 47.6 (C-19), 150.4 (C-20), 29.6 (C-21), 34.1 (C-22), 27.9 (C-23), 15.3 (C-24), 16.0 (C-25), 15.9 (C-26), 14.7 (C-27), 60.5 (C-28), 109.6 (C-29), 19.0 (C-30).

3-Hydroxy-11-ursen-28,13-olide (**3**) white amorphous powder; ESI-MS *m/z* 477.3 [M + Na]$^+$ (Calcd for $C_{30}H_{46}O_3$: 454.3); ^{1}H-NMR spectrum (400 MHz, pyridine-*d*$_5$): δ 5.98 (1H, d, *J* = 9.6 Hz, H-11), 5.62 (1H, d, *J* = 9.6 Hz, H-12), 3.41 (1H, dd, H-3), 1.19 (3H, s, Me-27), 1.17 (3H, s, Me-23), 1.15 (3H, s, Me-25), 0.96 (3H, s, Me-26), 0.87 (3H, s, Me-29), 0.82 (3H, s, Me-30), 0.75 (3H, s, Me-24). ^{13}C-NMR spectrum (100 MHz, pyridine-*d*$_5$): δ C: 36.5 (C-1), 25.8 (C-2), 77.9 (C-3), 39.4 (C-4), 55.0 (C-5), 19.2 (C-6), 31.9 (C-7), 41.9 (C-8), 53.3 (C-9), 38.5 (C-10), 133.6 (C-11), 129.3 (C-12), 89.5 (C-13), 42.1 (C-14), 27.8 (C-15), 23.1 (C-16), 45.1 (C-17), 60.4 (C-18), 38.1 (C-19), 40.2 (C-20), 30.9 (C-21), 31.4 (C-22), 28.3 (C-23), 16.1 (C-24), 19.2 (C-25), 19.1 (C-26), 15.8 (C-27), 179.3 (C-28), 17.8 (C-29), 18.1 (C-30).

Betulinic acid (**4**): white amorphous powder; ESI-MS *m/z* 455.3 $[M - H]^-$ (Calcd for $C_{30}H_{48}O_3$: 456.3); ^{1}H-NMR spectrum (400 MHz, pyridine-d_5): δ 4.91 (1H, brs, Hβ-29), 4.74 (1H, brs, Hα-29), 3.53 (1H, m, H-19), 3.40 (1H, t, *J* = 8.0 Hz, H-3), 2.68 (1H, m, H-13), 2.58 (1H, d, *J* = 12.0, H-16), 2.21 (1H, m, H-21), 1.77 (3H, s, Me-30), 1.19, 1.05, 1.03, 0.98, 0.80 (Me-23, Me-27, Me-26, Me-24, Me-25). ^{13}C-NMR (100 MHz, pyridine-d_5): δ C: 39.2 (C-1); 27.9 (C-2); 77.8 (C-3); 39.2 (C-4); 55.6 (C-5); 18.4 (C-6); 34.5 (C-7); 40.8 (C-8); 49.4 (C-9); 37.2 (C-10); 20.9 (C-11); 25.8 (C-12); 38.3 (C-13); 42.5 (C-14); 29.9 (C-15); 32.5 (C-16); 56.3 (C-17); 47.4 (C-18); 49.4 (C-19); 151.0 (C-20); 30.9 (C-21); 37.2 (C-22); 28.3 (C-23); 16.1 (C-24); 16.0 (C-25); 19.1 (C-26); 14.6 (C-27); 178.5 (C-28); 109.6 (C-29); 19.1 (C-30).

Oleanolic acid (**5**): white amorphous powder; ESI-MS *m/z* 479.3 $[M + Na]^+$ (Calcd for $C_{30}H_{48}O_3$: 456.3); ^{1}H-NMR spectrum (400 MHz, pyridine-d_5): δ 5.47 (1H, s, H-12), 3.42 (1H, dd, *J* = 5.6, 9.6 Hz, H-3), 3.31 (1H, dd, *J* = 10.4, H-18), 1.26 (3H, s, Me-27), 1.22, 1.03, 1.03, 1.00, 0.99, 0.93 (Me-23, Me-26, Me-30, Me-24, Me-29, Me-25). ^{13}C-NMR (100 MHz, pyridine-d_5): δ C: 38.8 (C-1); 28.0 (C-2); 78.0 (C-3); 39.3 (C-4); 55.7 (C-5); 18.7 (C-6); 33.2 (C-7); 39.6 (C-8); 48.0 (C-9); 37.3 (C-10); 23.6 (C-11); 122.4 (C-12); 144.8 (C-13); 42.1 (C-14); 28.2 (C-15); 23.7 (C-16); 46.6 (C-17); 41.9 (C-18); 46.4 (C-19); 30.9 (C-20); 34.2 (C-21); 33.2 (C-22); 28.7 (C-23); 16.5 (C-24); 15.6 (C-25); 17.5 (C-26); 26.1 (C-27); 180.4 (C-28); 33.2 (C-29); 23.7 (C-30).

Ursolic acid (**6**): white amorphous powder; ESI-MS *m/z* 479.3 $[M + Na]^+$ (Calcd for $C_{30}H_{48}O_3$: 456.3); ^{1}H-NMR spectrum (400 MHz, pyridine-d_5): δ 5.46 (1H, s, H-12), 3.43 (1H, dd, *J* = 6.8, 8.8 Hz, H-3), 2.62 (1H, d, *J* = 11.2, H-18), 1.22 (3H, s, Me-23), 1.21, 1.02, 1.02, 1.00, 0.97, 0.93 (Me-27, Me-26, Me-29, Me-24, Me-30, Me-25). ^{13}C-NMR (100 MHz, pyridine-d_5): δ C: 39.0 (C-1); 28.0 (C-2); 78.0 (C-3); 39.4 (C-4); 55.7 (C-5); 18.7 (C-6); 33.5 (C-7); 39.9 (C-8); 48.0 (C-9); 37.4 (C-10); 23.5 (C-11); 125.5 (C-12); 139.2 (C-13); 42.4 (C-14); 28.6 (C-15); 24.8 (C-16); 48.0 (C-17); 53.5 (C-18); 39.3 (C-19); 39.2 (C-20); 31.0 (C-21); 37.2 (C-22); 28.7 (C-23); 16.5 (C-24); 15.6 (C-25); 17.4 (C-26); 23.8 (C-27); 179.9 (C-28); 17.4 (C-29); 21.3 (C-30).

References

1. Prachayasittikul, S.; Saraban, P.; Cherdtrakulkiat, R.; Ruchirawat, S.; Prachayasittikul, V. New bioactive triterpenoids and antimalarial activity of *Diospyros rubra* Lec. *Excli J.* **2010**, *9*, doi:10.17877/DE290R-8933.
2. Pereira, S.I.; Freire, C.S.R.; Neto, C.P.; Silvestre, A.J.D.; Silva, A.M.S. Chemical composition of the epicuticular wax from the fruits of Eucalyptus globulus. *Phytochem. Anal.* **2005**, *16*, 364–369.
3. Ikuta, A.; Itokawa, H. Triterpenoids of Paeonia-Japonica Callus-Tissue. *Phytochemistry* **1988**, *27*, 2813–2815.
4. Seebacher, W.; Simic, N.; Weis, R.; Saf, R.; Kunert, O. Complete assignments of ^{1}H- and ^{13}C-NMR resonances of oleanolic acid, 18 alpha-oleanolic acid, ursolic acid and their 11-oxo derivatives. *Magn. Reson. Chem.* **2003**, *41*, 636–638.
